# Caring toward end of life through acute hospital and community partnerships: A scoping review

**DOI:** 10.1177/02692163241310692

**Published:** 2025-02-14

**Authors:** Joanna McIlveen, Catherine MacPhail, Mim Fox, Kerrie Noonan

**Affiliations:** 1 Faculty of the Arts, Social Sciences and Humanities, University of Wollongong, Wollongong, NSW, Australia; 2 Western NSW Local Health District, Dubbo, NSW, Australia; 3 School of Psychology, Western Sydney University, Penrith, NSW, Australia; 4 Public Health Palliative Care Unit, La Trobe University, Melbourne, VIC, Australia; 5 Death Literacy Institute, Dubbo, NSW, Australia

**Keywords:** Acute hospital, community, partnership, collaboration, work together, end of life, palliative care

## Abstract

**Background:**

Global health systems are currently socially and economically burdened. Public health palliative care is an approach to caring toward end of life that can create the innovative change needed to address this inequity. Guided by the Ottawa Charter for Health Promotion (1986), public health palliative care promotes collaboration among palliative care services, civic institutions, and communities to build capacity in all aspects of death, dying, caregiving, and bereavement. Despite growing evidence for the public health approach to palliative care, little is known about how acute hospitals and communities can work together to provide care toward end of life.

**Aim:**

To explore how acute hospitals and communities work together to provide care toward end of life.

**Design:**

Scoping review guided by Arskey and O’Malley framework.

**Data Sources:**

Scopus, Pubmed, CINAHL, and Informit as well as gray literature were searched. Citations were independently assessed against inclusion and exclusion criteria.

**Results:**

Of the six included studies and reports, a priori themes of creating supportive environments; strengthening community action; reorienting health services; developing personal skills and building healthy public policy from the Charter were well represented. Additional themes of communication and language, culture and risk were also identified. Educational, arts health, community engagement initiatives were explored as well as clinical tools, psychosocial interventions, and the No One Dies Alone (NODA) program.

**Conclusions:**

This review offers policymakers, hospitals, and practitioners a framework for implementing hospital-community partnerships toward end-of-life. Despite challenges in acute settings, these initiatives can enhance end-of-life experiences for patients and families.

**What is already known about the topic?**

- Public health palliative care approaches within hospice and community settings have been adopted throughout UK, Europe, Asia, and Australia.
- These approaches have the ability to build the capacity of communities in all aspects of death, dying, caregiving, and grief.
- It is unknown how acute hospitals can adopt public health approaches to palliative care through partnerships with community.

**What this paper adds**

- This review is a first of its kind using a public health palliative care framework to explore acute hospital and community partnerships toward end of life.
- It highlights key themes in partnership approaches toward hospital -community end of life programs, paying close attention to community engagement strategies and equitable power dynamics in order to re-orient hospitals and influence policy beyond localized initiatives.
- This review highlights the need for advocacy and policy translation to incorporate participatory methods that empower individuals and communities to work with hospitals, fostering cultural change and long-term transformation in care toward end of life.

**Implications for practice, theory or policy**

- Diverse interventions and programs indicate that healthcare practices should address the varied needs of patients and communities.
- Implementing community-based models and public health approaches can improve the quality of end-of-life care by fostering a more supportive environment for patients and families.
- Policy makers and health leaders can use the review to develop policies that support community and volunteer involvement toward end of life for the acute hospital setting, recognizing the value of enhancing patient and family well-being.
- Further research is needed to explore partnership models between the acute hospital and community toward end of life from the perspectives of health workers, hospital managers, and community members.

## Introduction

Dying in a hospital remains the most common experience of death in most high and middle income countries.^1–4^ With deaths across the OECD set to double over the next 25 years due to an aging population, hospitals are already experiencing the strain of chronic health conditions combined with limited financial and workforce resources.^2,5^ There is an imperative to critically examine existing approaches to care toward end of life and consider new approaches that may offer both individual and collective benefits.^6^

Internationally there have been multiple policy recommendations over the past 20 years calling for change and innovation in how we care for and support our aging, dying, and grieving.^1,7–9^ Public Health Palliative Care is an evidence-based complementary model for care toward end of life, which is gaining significant research and practitioner support.^10–12^

The World Health Organization’s Ottawa Charter for Health Promotion includes five principles and areas of action including creating supportive environments; strengthening community action; reorienting health services; developing personal skills and building healthy public policy.^13^ Stemming from these principles, the public health palliative care approach is a social model for end-of-life care firmly grounded in a social justice foundation.^10,14,15^ Key research exploring the public health palliative care approach from the UK and Europe highlights improved outcomes in health and well-being for individuals and families resulting from partnerships between health services and community.^14,16–20^

As the public health palliative care approach gains scholarly recognition there are still gaps in the literature querying its successful implementation. Much of the public health palliative care research to date has focused on the theoretical, conceptual, and process outcomes of public health palliative care, while very little literature exists evidencing collaborations and partnerships between acute hospitals and communities in providing care toward end of life.^9,10,14,21^ Against this background, the overall aim of this scoping review is to explore within existing literature how acute hospitals and communities work together to provide care toward end of life. A review of this nature will result in a comprehensive overview of existing hospital and community partnerships and collaboration, plus deepen our understanding of how public health palliative care approaches can reorient health systems to work in collaboration with the community.

For the purposes of this review, acute hospital refers to a healthcare setting where the focus is generally on diagnosis and treatment with a view to cure and discharge of the patient following sudden or urgent medical intervention.^22–24^ Community is defined as a group of people with diverse characteristics that may or may not be spatially connected, but who share common interests, concerns, or identities resulting in joint action.^25–28^ The terms partnership, collaboration, and working together are used interchangeably as reported in the literature.^29^ These terms describe a shared vision and commitment through mutual goal/s to improve capabilities, assets or resources based on a common purpose, need, or issue.^29–31^ The phrase “end of life” is generally a clinical term but for the purposes of this review it is used to describe individuals approaching their death, actively dying, or people who are bereaved following a death, due to the interconnectedness of death and bereavement.

## Methods

This scoping review was conducted according to the Arksey and O’Malley framework and informed by the PRISMA-ScR (Preferred Reporting Items for Systematic Reviews and Meta-Analyses Extension for Scoping Reviews).^32,33^ The review aimed to address the following lead and sub questions:

(1) How do acute hospitals and communities work together to provide care toward end of life?
(1a) What initiatives, interventions or programs have been undertaken and presented in the literature?
(1b) What are the key concepts and themes presented in the literature?

### Search strategy and information sources

The search strategy involved examining electronic databases, reference lists and reports from organizations.^32^ The authors agreed on four different scholarly databases (Scopus, Pubmed, CINAHL, and Informit) from when the review took place, January 2000– October 2023. The search was limited to English language and can be reviewed in Supplemental File 1. This review also included a search of grey literature from peak bodies Palliative Care Australia, Public Health Palliative Care International and the organization Compassionate Inverclyde. One Australian based report was emailed directly to a reviewer from a known acute hospital and community partnership initiative.

### Inclusion and exclusion criteria

Key inclusion criteria informed the design of the search strategy and included (1) adults of any gender, (2) any collaboration, partnership, or working together projects, initiatives or programs between the acute hospital and community carried out between 2000 and 2023, (3) all countries (4) original research articles (any method) and grey literature. All study designs were included due to niche subject matter. At the first stage of the screening and then the retrieval stage, the terms “hospice” and “health services” were included as the terms can be used interchangeably in the literature with the word hospital and the reviewers wanted to ensure suitable sources were not missed. The only exclusion criteria at this stage were literature involving babies, children, or adolescents.

**Figure 1.**
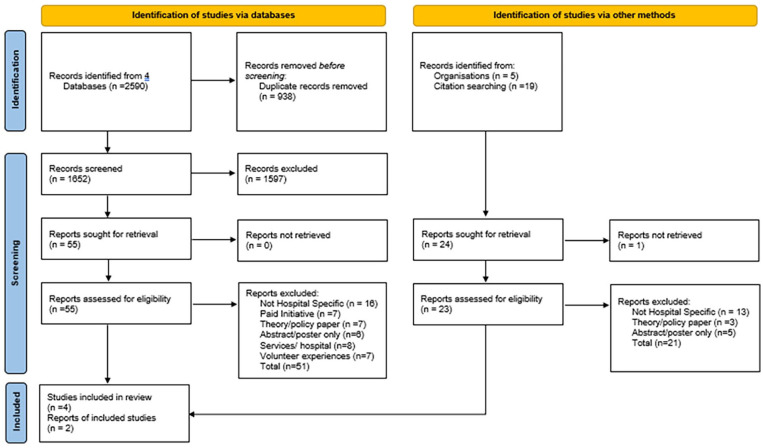
PRISMA flow chart of scoping review process.

Initially, all articles were imported into Endnote20 (Clarivate, PA, USA) where duplicate records were removed, and inclusion and exclusion criteria applied as demonstrated in the PRISMA flow chart in Figure 1.

### Search outcomes

The study selection process was implemented over two stages. The first stage involved reviewing document titles and keywords in Endnote 20 to determine which documents to retrieve and which to exclude based on the criteria. For the included documents, data was extracted into an MS Excel spreadsheet as recommended in the scoping review guidelines.^32^ Following this, a further review of the 78 documents was undertaken by reviewing the abstract to determine inclusion by two pairs of reviewers. In the MS Excel spreadsheet, documents were grouped based on type of reports: peer-reviewed, hand searched or grey literature. At this stage, an additional decision was made by three reviewers; 11 of the assessed documents were conference posters, abstracts, or abstracts in English, but the whole article was not. Although the documents met the criteria, they were excluded due to the infeasibility of translation and inadequate contribution to the richness of the review. Resulting from the specific interest in the acute hospital setting, reviewers researched each included acute hospital site to ensure they met the defined criteria of an acute hospital. Documents in which the setting of the work being undertaken was not explicitly between the acute hospital and community were excluded, namely “health service” or “hospice.” At this stage also, documents on palliative care volunteers’ motivations, experiences, or reflections were excluded as they didn’t meet the criteria of discussing initiatives, interventions, or programs.

### Charting the data

At the commencement of this scoping process, a draft charting form with standard characteristics was developed to assist data collection and organization of key themes from the documents included.^34^ A framework approach for this scoping review was implemented as it enables structure in data analysis and assistance with identifying themes and patterns.^35^ A priori themes based on the public health palliative care adoption of the Ottawa Charter for Health Promotion (creating supportive environments; strengthening community action; reorienting health services; developing personal skills and building healthy public policy) were chosen as this is foundational to the public health palliative care approach and to achieve populations scale impact in end of life, palliative care, and bereavement, working across all five areas of the charter is essential.^15^ Through the analytical process, the reviewers were able to actively construct themes outside of the Charter through a reflexive process of identifying, interpreting, and defining patterns within the data and embed these in the data chart.^36^ Using this method, information from the included documents was collated, summarized and reported, as detailed below.

## Results

As outlined in Figure 1, the database searches identified a total of 2590 articles. This was reduced to 1652 articles after duplicates were removed. Following the title and abstract screening of the documents, 55 were sought for retrieval and 24 hand searched and grey literature reports were retrieved for review. Following abstract assessment against the inclusion criteria, a total of six articles and reports were included in the review. Reasons for exclusion included articles that were not acute hospital specific (*n* = 29), theoretical or policy documents (*n* = 10), paid initiatives (*n* = 7), community services and hospitals partnerships (*n* = 8), volunteer experiences (*n* = 7) and abstract and conference presentations which did not provide an in-depth overview of the program provided (*n* = 11). Another two documents from Compassionate Inverclyde that mention the No One Dies Alone (NODA) program were also excluded as they lacked depth.^37,38^

### Characteristics of included studies

The articles and reports by Beers, Read, and Liu et al. all highlight the achievements of acute hospital and community partnerships including initiatives implemented, specific activities undertaken and their learnings.^39–41^ More specifically, Beers discusses an end-of-life coalition created to provide its community with quality end of life care.^39^ The document by Read is a project report outlining highlights for community engagement, greater socialized care, creative legacy arts health program, capacity building activities, and next steps for the project.^40^ Similarly, Liu et al. discuss the implementation of compassionate communities approaches in Taipei including experiences and preliminary outcomes.^41^ Documents by Barrie et al., Bradas et al., and Sardar explore the implementation process, lessons learned, and program expansion from the No One Dies Alone (NODA) programs being undertaken in the acute hospital setting in the United Kingdom and USA.^42–44^

Among the included articles and reports, three were from the last 5 years.^40,41,44^ The countries with the most identified articles and reports were the UK (2),^42,44^ USA (2),^39,43^ followed by Australia, (1)^40^ and Taiwan (1).^41^ Three of the six documents also specifically mention the public health palliative care approach, demonstrating the significance of the approach when considering an acute hospital and community partnership in providing care toward end of life.^40–42^

#### Initiatives, interventions or programs

Acute hospitals and communities undertake a variety of initiatives, interventions, and programs in caring toward end of life as outlined in Table 1. Education and capacity building activities included educational booklets,^39^ educational initiatives for health care staff and volunteers,^39–44^ and workshops and conferences undertaken within the collaboration.^40,41^ Arts Health programs featured, including the Festival of life, art exhibitions, film, drawing, and essay competitions,^41^ as well as visits from choirs and orchestras and a creative legacy program.^40^ Life Issues Café,^41^ round table meetings,^39,41,44^ performances and book club,^41^ garden clubs and morning teas,^40^ were examples of community engagement with citizens, councils, schools, and the arts community. Clinical care tools in pain and symptom management plus advance care planning support evolved from the partnerships,^39,41^ as did psycho-social interventions such as grief support groups,^39^ memorial activities,^41^ a bucket list project,^41^ and social network mapping and ward social dinners.^40^

**Table 1.** Intervention, initiatives, and programs across the documents.

Intervention/ Initiatives/ Programs	Beers (2000)	Bradas et al. (2014)	Barrie et al. (2018)	Read (2019)	Sardar (2019)	Liu et al. (2022)
Capacity building/ Educational	x	x	x	x	x	x
Arts health				x		x
Community engagement	x	x	x	x	x	x
Clinical tools	x					x
Psycho-social interventions	x	x	x	x	x	x
No one dies alone program (specifically)		x	x		x	

Three of the six documents in this review specifically discuss the No One Dies Alone program (NODA). An end-of-life companionship program, NODA originated in the USA and is a hospital led volunteer-based initiative.^43^ Volunteers are specially recruited and trained to provide comfort to dying individuals without family or friends present.^44^ If it aligns with the patient’s wishes, NODA volunteers provide comfort by playing music, reading, holding their hand, creating a soothing environment, and offering their presence during the final moments.^44^ Some programs also provide respite to vigilling families.^42,44^

#### Key concepts and themes

As discussed previously, a priori themes based on the Ottawa Charter for Health Promotion (1986) were selected to provide a framework for the scoping review. At the core of the charter is the understanding that health care should be participatory, not something “*we do to others*” instead something “*we do with others.”*^45^ Theoretically, the way in which acute hospitals and communities work together to provide care toward end of life connects to this principle of undertaking activities with each other. Despite not all the documents explicitly taking a public health palliative care approach, all five of the a priori themes based on the Ottawa Charter were identified throughout the six documents. Below we have defined the themes to systematically extract the data from the documents.

### Strengthen community action

Through the provision of education and information on health, dying and death, a community can actively pursue priorities, strategies, and policies enabling them to live well and die well through collective organization and action.^15^ This Ottawa principle is firmly centered around the empowerment of communities and community taking ownership of death, dying, and bereavement.^13^ Liu et al. discusses how empowering community is an effective strategy not only to build capacity and take ownership but also enhances support provided to individuals and families who are experiencing loss, with the hope of being able to integrate the loss into their everyday lives.^41^

Using existing community resources such as people and networks, the ability to strengthen public participation in death, dying, and bereavement can enable greater community action.^13^ All documents discuss a multifaceted approach to strengthening community action such as forming broad alliances, activating community groups, using media channels, social media and creative arts initiatives to capture a larger audience.^39–44^ This in turn raises awareness and contributes to providing education to community members. Barrie et al., Liu et al., and Sardar examine how community action can be strengthened through storytelling.^41,42,44^ By igniting the community’s imagination through an emotive personal experience, there is great potential to galvanize collective action. Bradas et al. and Read also discuss ways in which they strengthen community action by leveraging an individual’s strengths, skills, and resources to enhance the project.^40,43^ Agency such as this fosters a deeper sense of ownership and investment in community actions and programs and empowers individuals to create change.

### Create supportive environments

The ability to create supportive environments through the provision of social supports, both personal and community, is grounded in the notion of strengthening existing relations and developing new ones which ultimately enhances a sense of well-being.^46^ Throughout all six included documents, creating supportive environments is strongly represented. Read, Liu et al. and Barrie et al. discuss multiple strategies to increase participation and involvement from service users, community groups, and health professionals resulting in the enhanced experience of participants.^40–42^ Sardar, Bradas et al. and Beer demonstrate the creation of supportive environments within their programs from a relationship building perspective, highlighting the importance of human connection.^39,43,44^ Barrie et al. discuss how shared values among volunteers foster connections and friendships within the volunteer group and Bradas et al. highlight the role of supportive environments in volunteer retention.^42,43^

### Develop personal skills

All six of the documents discuss how their programs enhance life skills through sharing, education and information that promotes the personal and social development of communities.^13^ By developing personal skills in how to support and care for the dead, dying, and grieving through a process of interpersonal reorientation, individuals, and communities can play a greater role in caring toward end of life.^46^ The NODA documents by Barrie et al., Sardar and Bradas et al. directly discuss how volunteers develop practical personal skills in end-of-life care as they companion people who are dying.^42–44^ Both Read and Liu et al. discuss the impact of their intergenerational programs on the ability to develop personal skills in caring toward end of life such as shared learning of life experiences, improved communication, knowledge exchange, and interpersonal growth.^40,41^ All six documents explore personal empowerment from increased knowledge and skills in death, dying, and bereavement which subsequently enables us to make informed choices and have greater control over issues relating to end of life.

### Reorient health services

Re-orientating the health system requires individuals, community groups, health services, and government to work together and refocus on the total needs of the individual and move beyond providing only clinical and curative services.^13^ The Ottawa Charter emphasizes that for health services to be reoriented, care should also encompass preventative measures, health promotion, and consideration of the social determinants of health. Read and Liu et al. both discuss the integration of health promotion into their approaches and programs,^40,41^ Liu et al. particularly emphasizing it as central to the work they do.^41^ Liu et al. acknowledges the importance of prevention in reference to public health approaches but does not expand on how their programs enable preventative measures.^41^ Following our analysis, it became apparent that the social determinants of health were not a strong feature of the included documents, highlighting a gap when it comes to reorienting health services. Liu et al. does mention vulnerability and low economic status in reference to the social determinants of health but does not expand upon this point.^41^

Within this review however, all of the six included articles discussed collaboration as a key factor when working to reorient health services and encourage a shared approach for caring toward end of life.^39–44^ Pursuing “health” collaboratively, a goal of the Ottawa Charter, also involves education, training, and research.^13^ For palliative care services specifically, reorientation also involves genuine collaboration and partnerships with local councils, local faith groups, schools, community groups, and volunteer groups.^46^ Creating opportunities for pathways between the health sector and broader economic, social, cultural and political systems enables increased support for individuals and communities with palliative care needs and lessens the load for individual organizations.^13^ Liu et al., Read and Sardar discuss how working together decreases the silo mentality of organizations, particularly health services, which can ultimately reduce the workload pressure on staff.^40,41,44^ Beers also discusses the importance of research and evaluation when considering how to reorient health services to highlight the additional benefits of collaborative end of life approaches to health services.^39^ Read, Sardar and Liu et al. mention research approaches they have incorporated into their programs to demonstrate efficacy but do not explicitly discuss how this evidence can be used to reorient the health service.^40,41,44^

### Build healthy public policy

According to Kellehear, building public policies that support dying, death, loss and grief, and challenge existing death denying health policies and attitudes is fundamental to creating change.^46^ The development of healthy end of life policy is a concern and responsibility of each sector and at every level of government whereby policies “identify obstacles to dying well and remove barriers that contribute to poor end of life outcomes.”^15^ All six articles within this scoping review speak to the influences the programs, projects, or initiatives have had at micro and meso policy levels such as ward,^40,42^ hospital,^41,43^ health service,^39^ and local council area.^39–41^ The included documents do not however address current macro level policies that can contribute to the implementation of the programs on a state or national level. Liu et al. and Read did however report on future plans stemming from their work, including approaches to enhance the reach of the programs at a macro level, such as visits to the ward from policy makers, further funding from state-based organizations, growing partnership opportunities, and using the successes of the programs to garner greater buy-in from more government agencies.^40,41^

### Themes outside of the Ottawa Charter for health promotion

#### Communication and language

Across the documents, the theme of communication and language is critical when considering hospital and community partnerships in caring toward end of life. Both Liu et al. and Beers discuss cohesive and effective communication as being essential for successful outcomes in partnerships.^39,41^ Beers emphasizes the need for a common language in hospital-community partnerships to reduce ambiguity,^39^ while Barrie et al. discusses how language evolves with community needs.^42^ Sardar reflects on the use of sensitive communication as an inclusion in the training for volunteers with the NODA Program,^44^ and Bradas et al. and Barrie et al. highlight the importance communication has when promoting the NODA service within and outside of the hospital setting.^42,43^ Creative approaches to communication taken in the projects by Read demonstrate how they allow the community and staff to engage in diverse ways outside of their routine activities.^40^

#### Culture

Acute hospital and community partnerships must be aware of how culture impacts, not only on the patients and families and those involved within the partnership, but also the culture of partnership itself. Beers describes having to counter a culture in which individuals and the community more broadly are reluctant to talk about death and dying by developing a resource to serve as a catalyst to address the taboo of death.^39^ Barrie et al. reflect on the values of kindness and compassion embedded in the culture of the NODA program,^42^ while Read reports that partnerships have the potential to contribute to cultural safety and inclusion within the hospital environment and demonstrate an ability to create rich and meaningful experiences from acute hospital community partnerships if there is encouragement and support enabling an expression of culture.^40^ Finally, Liu et al. discusses how the experience of death, dying, and bereavement occurs within a cultural framework and is governed through cultural norms and traditions.^41^

#### Risk

Articles by Bradas et al., Barrie et al. and Sardar all highlight the balance that is required between the acute hospital and the community partnership, to ensure both sides are aware of and protected against the risks that may result from the activities undertaken.^42–44^ Barrie et al. report that to ensure the NODA program runs smoothly it adheres to necessary requirements for volunteers whilst avoiding overregulation and formalities which could hinder the kindness of volunteers.^42^ Both Sardar and Bradas et al. are explicit in how they manage risk and discuss volunteer screening as essential to ensure success of the program.^43,44^

## Discussion

### Main findings of the study

This scoping review examined six documents that explore how acute hospitals and community can work together to provide care toward end of life. It highlights the diverse approaches policy makers, health service managers, and practitioners can take to increase community engagement in end of life care. The need for genuine community involvement to ensure sustainable change is crucial when establishing these partnership approaches, but hospitals and health services must also reduce the barriers for community participation and rebalance power if they want community to play an equal role in providing care toward end of life.

When considering the interventions, initiatives, and programs undertaken between the hospital and the community, (as highlighted in Table 1) they all encompass community engagement strategies in end-of-life care with the aim of building death and grief literacy.^47,48^ As Sallnow and Paul describe,^49^ community engagement is a partnership approach services and communities can take using diverse methods to raise awareness, build capacity, and address gaps related to death, dying, and bereavement. According to Noonan, to create sustainable cultural change in the community when it comes to end-of-life matters, understanding when programs are community engagement or community development is vital.^47^ Community engagement is concerned with informing and consulting takes a passive tone such as health services providing information to assist communities or obtain feedback from communities on options and decisions.^50^ At the other end of this spectrum and informed by a community development framework, is the enactment of community engagement which enables bottom up approaches encouraging participation, co-design, and empowerment strategies when designing programs that are the real catalyst for social change and transformation.^47,49^ The documents from Liu et al. and Read mention community development, participation, and empowerment, however no specific detail is described in how they used these approaches.^40,41^ Barrie et al. briefly mention assets-based approaches and co-production, other terms for community development methods, but again do not reflect further on this in the document.^42^ The remaining three documents do not describe community engagement strategies at all. Subsequently, it is challenging to establish if these programs will create sustainable social change or demonstrate the long-term success of the hospital and community working together to provide care toward end of life. Moving forward, when hospitals and communities work together on developing and implementing these types of programs, ensuring that there is a process that includes genuine community engagement would be essential to embed sustainable long-term change. This approach is consistent with the values and principles of the Ottawa Charter and promotes the Charter’s call for individual autonomy over one’s wellbeing as well as comprehensive, participatory and empowering actions to foster healthier, more resilient communities.^13^

Communication and language is paramount in fostering successful partnerships, ensuring that they function efficiently and effectively.^51^ According to literature, effective communication hinges on key components such as nonjudgment, empathy, authenticity, and collaboration.^52^ By embodying these principles, partnerships can develop and thrive, fostering sustainability, and success. Language also holds significant sway, shaping our perceptions and behaviors.^53,54^ In discussions surrounding death, dying, and grief, clear and direct communication, rather than euphemisms, is crucial, especially within hospital settings.^55^ However, this clarity may not always extend to broader community conversations and the cultural norms of the community must be considered as cultural context further influences experiences of death and bereavement and is governed by norms and traditions.^56^ Additionally, in partnership approaches, managing risk can pose a barrier, deterring individuals and communities from engaging in partnerships with institutions that prioritize risk-averse management approaches.^57,58^ Thus, addressing these factors within partnerships is crucial for fostering collaboration, understanding, and ultimately, success.

When considering how acute hospitals and communities work together to provide care toward end of life, consideration and discussion about power and partnerships is essential, particularly when applying a public health palliative care lens. A key element of partnership dynamics is power and there is often a large power differential between large organizations and the community. According to Rosenberg, new public health approaches identify equal partners as ones in which power and expertise of service providers is challenged and renegotiated.^59^ A true partnership is one where each group contributes their own expertise and knowledge and both communities and services share and hold equal position in the partnership. Further, true partnerships integrate the mutual goals of the institution (in this review the institution being the hospital) and community to achieve a shared vision through processes such as co-design.^47^ Given this perspective, if the power and decision-making rests solely with the health service, hospital, or organization then it is not an equal partnership. When applying this lens to the NODA programs discussed in this scoping review, it can be argued that NODA is a service lead model of partnership and although the community (volunteers) hold very little power, this is not a deterrence to support the initiative. Quite possibly, it is not a partnership at all, rather service-recipient relations, service provision, or patient care utilizing community resources to complement existing health care.^19,60^ The NODA program does however meet the criteria for the hospital and community working together to provide care toward end of life and, although not a true partnership in the eyes of the public health palliative care approach, a valuable, compassionate and valid contribution to caring at end of life. If acute hospitals can offer a companioning program at end of life this would be a welcomed addition to patient care. For the documents by Beers, Read and Liu et al. it is difficult to comment on power dynamics within the partnerships, projects and initiatives as it has not been reported on.^39–41^

A significant finding of this review is that half of the included documents discuss the NODA program. This prominence is likely due to NODA’s well-defined scope and focus on individual outcomes, which make it easier to research and publish compared to other acute hospital-community initiatives.^61^ Additionally, the controlled institutional setting of a hospital streamlines study design, data collection, and analysis, thereby reducing logistical barriers and enabling quicker insights. NODA studies don’t require community-wide metrics, unlike public health palliative care programs that demand complex, long-term social impact measures.^49,62^ This clear, structured approach makes NODA research more accessible and publishable which speaks to the significant representation of the literature in this review.

The question remains however, is there such a thing as a “true” partnership when large healthcare organizations such as an acute hospital are involved? Sallnow demonstrates that partnership between community and hospice does exist when values of reciprocity, trust and respect are imbued resulting in the community feeling a sense of ownership of the program.^19^ Aoun and colleagues evidence the ability of regional health services and community to partner in caring toward end of life through a compassionate communities connector model.^63,64^ This model serves to increase the practical and social supports to improve social connectedness for people who are at end of life.^63^ From a service provider perspective, the connectors program is seen as providing something extra and complementary to existing health care provision and this perspective highlights the power sharing that is inherit in the model, enabling the success of the partnership.^58,63^ Though the authors do not explicitly discuss it, the articles strongly suggest that community partners feel a deep sense of ownership over the program, which in turn helps to embed values of reciprocity, trust, and respect within its structure.^61,63^

Reorienting professional power in the acute hospital setting according to this scoping review proves to be more challenging. The lack of literature for acute hospital and community partnerships may be due to the traditional ingrained hierarchical structure within the hospital setting, making it difficult to integrate community approaches into hospital practices.^59^ It may also be an outcome of the prevailing biomedical dominance which champions expertise and control over the dying as opposed to genuinely valuing the skills, knowledge, and experience of community.^58^ Environments such as the acute hospital may find it challenging to relinquish some of that power by respecting and working with the whole community to care for individuals toward end of life. Additionally, the acute hospital system is a complex regulatory environment which, in and of itself, hinders the ability to engage with the community effectively. From the results of this scoping review, reorienting acute hospitals to work in partnership with community is a huge challenge for policy makers, hospital managers, health care clinicians, and the community more broadly. Further research is needed to determine if and how an acute hospital and community can work together to provide care toward end of life. Additionally, does “working together” embody a true and equal partnership or something else entirely?

Finally, this scoping review highlights the success that has been achieved in building healthy public policy on both micro and meso policy levels, yet the lack that still exists when including the macro (or policy) level. Often, hospital or community-based programs can serve as models or pilots to demonstrate the effectiveness of certain approaches or interventions in improving outcomes or reducing costs. Policymakers may use evidence from these programs to inform the development of broader policies aimed at replicating successful strategies across healthcare systems or regions.^65,66^ Although community driven, ground up approaches can influence policy at both local and national levels and also reflects the inherit values that community embodies, of the documents included in the scoping review, there has been no translation of the small-scale policies to larger ones, indicating that there is still further work to be done.^9^

### What this study adds

This study adds valuable insights into the complexities and challenges of creating sustainable partnerships between acute hospitals and communities in caring toward end of life. It highlights the practice possibilities of recent hospital-community collaborations which can be adapted to suit the setting, yet acknowledges the inherit importance of health promotion, power, culture, and communication when establishing these programs. It demonstrates that while community-led models like compassionate communities effectively foster social connection and support, similar approaches are difficult to integrate into hierarchical hospital settings and subsequently NODA- type programs may be more suitable for this setting. This study further contributes by emphasizing the need for broader policy translation and advocating for co-designed, participatory methods to bridge gaps in end-of-life care, suggesting areas for future research and development.

### Limitations of the study

Due to feasibility, only English language articles and documents were included and programs that may have been reported on in non—English languages are unable to contribute to the discussion. Since the inclusion criteria focused solely on acute hospitals, programs in hospices, such as befriending initiatives, compassionate neighbors, culturally competent community hospice programs, and teen volunteer programs, were excluded as well as the work being undertaken in health services.^19,20,61,63,67–70^ An additional limitation of this scoping review was the inability to include a consultation exercise with experts, potentially creating a gap in the literature, particularly in grey literature such as unpublished documents relevant to this review. This review however is a first of its kind in using a public health palliative care framework to explore acute hospital and community partnerships in caring toward end of life. It has highlighted existing gaps in the literature and enabled greater thinking about partnerships and power in collaborative work being undertaken by healthcare services and the community. In a practical sense, it has also highlighted examples of programs that hospitals and communities could look to implement in their own spaces when exploring possible compassionate community approaches to caring toward end of life.

## Conclusion

The Ottawa Charter for Health Promotion is crucial in examining hospital and community partnerships in caring toward end of life, with its action areas prominently featured in all six included documents. Additional themes relating to communication and language, culture and risk were also identified from the analysis. The review’s findings are important for health services, clinicians, and communities to reflect upon when embarking end-of-life partnership projects whilst contemplating power sharing and sustainable social change relating to all aspects of death, dying and bereavement in our community.

## Supplemental Material

sj-docx-1-pmj-10.1177_02692163241310692 – Supplemental material for Caring toward end of life through acute hospital and community partnerships: A scoping review Supplemental material, sj-docx-1-pmj-10.1177_02692163241310692 for Caring toward end of life through acute hospital and community partnerships: A scoping review by Joanna McIlveen, Catherine MacPhail, Mim Fox and Kerrie Noonan in Palliative Medicine

## References

[1] Sallnow L Smith R Ahmedzai SH , et al.; Lancet Commission on the Value of Death. Report of the lancet commission on the value of death; bringing death back to life. Lancet 2022; 39(10327): 837–884. 10.1016/S0140-6736(21)02314-X PMC8803389 35114146

[bibr2-02692163241310692] OECD. Time for better care at the end of life. OECD health policy studies. Paris: OECD Publishing, 2023.

[bibr3-02692163241310692] Adair T. Who dies where? Estimating the percentage of deaths that occur at home. BMJ Glob Health 2021; 6(9): 1-10. 10.1136/bmjgh-2021-006766 PMC8420738 34479953

[4] Broad JB Gott M Kim H , et al. Where do people die? An international comparison of the percentage of deaths occurring in hospital and residential aged care settings in 45 populations, using published and available statistics. Int J Public Health 2013; 58(2): 257–267. 22892713 10.1007/s00038-012-0394-5

[5] Swerissen H Duckett S. Dying well. Report, Grattan Institute, Victoria, 2014.

[6] Noonan K. Renegade stories: a study of deathworkers using social approaches to dying, death and loss in Australia. PhD Thesis, Western Sydney University, Australia, 2018.

[7] Rosenberg J. Whose business is dying?: death, the home and palliative care. Cult Stud Rev 2011; 17(1): 15–30.

[bibr8-02692163241310692] Nous Group. Final report: compassionate communities feasibility study, https://palliativecare.org.au/wp-content/uploads/dlm_uploads/2018/09/Compassionate-Communities-Final-Report-min.pdf (2018, accessed 3 March 2022).

[9] Sallnow L. Prevention and harm reduction. In: Abel J Kellehear A (eds.) Oxford textbook of public health palliative care. Oxford: Oxford University Press, 2022, pp.73–84.

[10] Collins A Brown JEH Mills J , et al. The impact of public health palliative care interventions on health system outcomes: a systematic review. Palliat Med 2021; 35(3): 473–485. 33353507 10.1177/0269216320981722

[bibr11-02692163241310692] Abel J Kellehear A. Oxford textbook of public health palliative care. Oxford: Oxford University Press, 2022.

[12] Bakelants H Vanderstichelen S Chambaere K , et al. Researching compassionate communities: identifying theoretical frameworks to evaluate the complex processes behind public health palliative care initiatives. Palliat Med 2023; 37(2): 291–301. 36576313 10.1177/02692163221146589

[13] World Health Organization. Ottawa charter for health promotion. In: First international conference on health promotion,Ottawa, 21 November 1986. Geneva: World Health Organization.

[14] Abel J Kellehear A Karapliagou A. Palliative care—the new essentials. Ann Palliat Med 2018; 7(Suppl 2): S3–14. 10.21037/apm.2018.03.04 29764169

[15] Grindrod A. Health promotion and palliative care. In: Abel J Kellehear A (eds) Oxford textbook of public health palliative care. Oxford: Oxford University Press, 2022, pp.146–154.

[16] Molina EH Nuño-Solinis R Idioaga GE , et al. Impact of a home-based social welfare program on care for palliative patients in the Basque Country (SAIATU Program). BMC Palliat Care 2013; 12: 3. 23363526 10.1186/1472-684X-12-3 PMC3576230

[bibr17-02692163241310692] Nuño-Solinís R Herrera Molina E Librada Flores S , et al. Care costs and activity in the last three months of life of cancer patients who died in the Basque Country (Spain). Gac Sanit 2017; 31(6): 524–530. 27707518 10.1016/j.gaceta.2016.06.005

[bibr18-02692163241310692] Cronin CE Franz B Garlington S. Population health partnerships and social capital: Facilitating hospital-community partnerships. SSM Popul Health 2021; 13: 100739. 33537403 10.1016/j.ssmph.2021.100739 PMC7841352

[bibr19-02692163241310692] Sallnow L. Collective social capital: a study of new public health and end of life care. PhD Thesis, University of Edinburgh, UK, 2017.

[20] Walshe C Algorta GP Dodd S , et al. Protocol for the End-of-Life Social Action Study (ELSA): a randomised wait-list controlled trial and embedded qualitative case study evaluation assessing the causal impact of social action befriending services on end of life experience. BMC Palliat Care 2016; 15: 60. 27412459 10.1186/s12904-016-0134-3 PMC4944471

[21] Horsfall D Leonard R Noonan K , et al. Working together–apart: exploring the relationships between formal and informal care networks for people dying at home. Prog Palliat Care 2013; 21(6): 331–336.

[22] Australian Institute for Health and Welfare. Establishment type. Establishment—establishment type, sector and services provided code AN.N{.N} (aihw.gov.au) (2018, accessed 23 August 2023).

[bibr23-02692163241310692] Australian Commission on Safety and Quality in Health Care. Safety and quality of end-of-life care in acute hospitals: a background paper. Sydney: ACSQHC, 2013.

[24] Hirshon JM Risko N Calvello EJ , et al. For the Acute Care Research Collaborative at the University of Maryland Global Health Initiative. Acute care research collaborative at the University of Maryland Global Health Initiative. Health systems and services: the role of acute care. Bull World Health Organ 2013; 91(5): 386–388. 23678202 10.2471/BLT.12.112664 PMC3646345

[25] Spreckley M Kellehear . The ‘new’ public health: the theoretical origins of public health palliative care. In: Abel J Kellehear A (eds) Oxford Textbook of Public Health Palliative Care. Oxford: Oxford University Press, 2022, pp.52–61.

[bibr26-02692163241310692] Wallerstein N Duran B Oetzel JG , et al. (eds). Community-based participatory research for health: advancing social and health equity. 3rd ed. San Francisco, CA: Jossey-Bass, 2018.

[bibr27-02692163241310692] World Health Organisation. The 7th global conference on health promotions, Nairobi, Kenya, 2009.

[28] MacQueen KM McLellan E Metzger DS , et al. What is community? An evidence-based definition for participatory public health. Am J Public Health 2001; 91(12): 1929–1938. 11726368 10.2105/ajph.91.12.1929 PMC1446907

[29] Carnwell R Carson A. The concepts of partnership and collaboration. In: Carnwell R Buchanan J (eds) Effective practice in health, social care and criminal justice: a partnership approach. Maidenhead, UK: Open University Press, 2009, pp.3–21.

[bibr30-02692163241310692] Mayan M Pauchulo AL Gillespie D , et al. The promise of collective impact partnerships. Community Dev J 2020; 55(3): 515–532.

[31] Thiel M. SDG17 - partnerships for the goals: strengthening implementation through global cooperation. Bingley: Emerald Publishing Limited, 2019.

[32] Arksey H O’Malley L. Scoping studies: towards a methodological framework. Int J Soc Res Methodol 2003; 8(1): 19–32.

[33] Tricco AC Lillie E Zarin W , et al. PRISMA extension for scoping reviews (PRISMA-ScR): checklist and explanation. Ann Intern Med 2018; 169(7): 467–473. 30178033 10.7326/M18-0850

[34] Archibald D Patterson R Haraldsdottir E , et al. Mapping the progress and impacts of public health approaches to palliative care: a scoping review protocol. BMJ Open 2016; 6(7): e012058. 10.1136/bmjopen-2016-012058 PMC4947716 27417201

[35] Green J Thorogood N (eds). Qualitative methods for health research. 4th ed. London: Sage, 2018.

[36] Braun V Clarke V. Using thematic analysis in psychology. Qual Res Psychol 2006; 3(2): 77–101.

[37] Barrie K Miller E O’Brien M. Compassionate inverclyde: evaluation report: a deeper dive, https://ardgowanhospice.org.uk/wp-content/uploads/2018/12/CI-evaluation_-Deeper-Dive.pdf (2018, accessed 21 March 2022).

[38] Barrie K Miller E O’Brien M. Compassionate inverclyde: evaluation summary, https://ardgowanhospice.org.uk/wp-content/uploads/2018/12/CI-evaluation_-summary.pdf (2018, accessed 21 March 2022).

[39] Beers W. When the community cares. Health Prog 2000; 81(1): 43–45. 11067070

[bibr40-02692163241310692] Read N. Ward without walls project report. Liverpool Hospital Palliative Care & The Groundswell Project, Sydney, 2019.

[bibr41-02692163241310692] Liu CJ Huang SJ Wang SS. Implementation of compassionate communities: the Taipei experience. Healthcare 2022; 10(1): 1–10. 10.3390/healthcare10010177 PMC8776212 35052340

[bibr42-02692163241310692] Barrie K Miller E O’Brien M. Compassionate inverclyde voices: the narrative from a local perspective, https://ardgowanhospice.org.uk/wp-content/uploads/2018/12/CI-evaluation-_Voices-Narrative.pdf (2018 accessed 21 March 2022).

[bibr43-02692163241310692] Bradas C Bowden V Moldaver B , et al. Implementing the ‘No One Dies Alone’ program: process and lessons learned. Geriatr Nurs 2014; 35(6): 471–473. 25457287 10.1016/j.gerinurse.2014.10.005

[44] Sardar S. No one should die alone: volunteer support for patients dying in hospital. Nurs Times 2019; 115(12): 34–36.

[45] Kellehear A. Compassionate cities. Public health and end of life care. London: Routledge, 2005, pp.25.

[46] Kellehear A. Health promoting palliative care. Oxford: Oxford University Press, 1999.

[47] Noonan K. Participatory relations. In: Abel J Kellehear A (eds) Oxford textbook of public health palliative care. Oxford: Oxford University Press, 2022, pp.95–102.

[48] Breen LJ Kawashima D Joy K , et al. Grief literacy: a call to action for compassionate communities. Death Stud 2022; 46(2): 425–433. 32189580 10.1080/07481187.2020.1739780

[49] Sallnow L Paul S. Understanding community engagement in end-of-life care: developing conceptual clarity. Crit Public Health 2014; 25(2): 231–238.

[50] Rosenberg J Sheldrake M. Community engagement strategies: in the too-hard basket? In: The 13th Australian palliative care conference, Melbourne, Australia, 2015.

[51] Partnership Brokers Association. Brokering better partnerships by investing in the partnering process, https://partnershipbrokers.org/w/wp-content/uploads/2021/02/Brokering-Better-Partnerships-Handbook.pdf (2019, accessed 19 May 2023).

[52] McCormack B McCance T. (eds) Person-centred practice in nursing and healthcare. Hoboken, NJ: John Wiley & Sons, Inc, 2016.

[53] Tucker KL Steele FB. Patient choice at the end of life: getting the language right. J Leg Med 2007; 28(3): 305–325. 17885903 10.1080/01947640701554427

[54] O’Connor M Payne S . Discourse analysis: examining the potential for research in palliative care. Palliat Med 2006; 20(8): 829–834. 17148538 10.1177/0269216306072348

[55] Barlet MH Barks MC Ubel PA , et al. Characterizing the language used to discuss death in family meetings for critically ill infants. JAMA Netw Open 2022; 5(10): e2233722. 10.1001/jamanetworkopen.2022.33722 PMC9535532 36197666

[56] Klass D Chow A. Culture and ethnicity in experiencing, policing, and handling grief. In: Neimeyer RA Harris DL Winokuer HR , et al. (eds) Grief and bereavement in contemporary society: bridging research and practice. New York, NY: Routledge, 2011, pp.341–353.

[57] Sallnow L Richardson H. Volunteering and community. In: Scott R Howlett S (eds) The changing face of volunteering in hospice and palliative care. Oxford: Oxford University Press, 2018﻿; 185–196.

[58] Noonan K Rumbold B Aoun SM. Compassionate community connectors: a distinct form of end-of-life volunteering. Prog Palliat Care 2023; 31(1): 1–10.

[59] Rosenberg JP. Early intervention. In: Abel J Kellehear A (eds) Oxford textbook of public health palliative care. Oxford: Oxford University Press, 2022, pp.85–103.

[60] Sallnow L Bunnin A Richardson H. Community development and hospices: A national UK perspective. In: Wegleitner K Heimerl K Kellehear A (eds) Compassionate communities: case studies from Britain and Europe. Oxon: Routledge, 2015; 1–14.

[61] Greenhalgh T Raftery J Hanney S , et al. Research impact: a narrative review. BMC Med 2016; 14: 78. 27211576 10.1186/s12916-016-0620-8 PMC4876557

[62] Sawyer J Sallnow L. The evidence for the effectiveness of a public health palliative care approach. In: Abel J Kellehear A (eds) Oxford textbook of public health palliative care. Oxford: Oxford University Press, 2022, pp.222–229.

[63] Aoun SM Abel J Rumbold B , et al. The compassionate communities connectors model for end-of-life care: a community and health service partnership in Western Australia. Palliat Care Soc Pract 2020; 14: 2632352420935130. 10.1177/2632352420935130 PMC7333490 32656530

[64] Aoun SM Rosenberg J Richmond R , et al. The compassionate communities connectors programme: experiences of supported families and referring healthcare providers. Palliat Care Soc Pract 2023; 17: 26323524231173705. 10.1177/26323524231173705 PMC10184225 37197223

[65] Zelmer J. Beyond pilots: scaling and spreading innovation in healthcare. Healthc Policy 2015; 11(2): 8–9. 26742111 PMC4729277

[66] Winter MD. Reshaping health care governance using pilot projects as public policy implementation instruments. Int Rev Public Policy 2020; 2(3): 317–341.

[67] Townsend A March AL Kimball J. Can faith and hospice coexist: is the African American church the key to increased hospice utilization for African Americans? J Transcult Nurs 2017; 28(1): 32–39. 26297709 10.1177/1043659615600764

[bibr68-02692163241310692] Mascagni G. Collaboration and emotions to the test: the experience of FILe volunteers in hospices. Acta Biomed 2016; 87(4-S): 60–70. 27874845

[bibr69-02692163241310692] Reese DJ. Proposal for a university-community-hospice partnership to address organizational barriers to cultural competence. Am J Hosp Palliat Care 2011; 28(1): 22–26. 20508242 10.1177/1049909110370744

[70] Letizia M Zerby B Hammer K , et al. The development of a hospice junior volunteer program. Am J Hosp Palliat Care 2000; 17(6): 385–388. 11886039 10.1177/104990910001700608

