# Supplementary material for: Caring toward end of life through acute hospital and community partnerships: A scoping review
Source: Palliat Med. 2025 Feb 14;39(3):346–57. doi: 10.1177/02692163241310692 (PMC11877981; doi:10.1177/02692163241310692)
Supplement: sj-docx-1-pmj-10.1177_02692163241310692 – Supplemental material for Caring toward end of life through acute hospital and community partnerships: A scoping review [file sj-docx-1-pmj-10.1177_02692163241310692.docx]

Supplementary file 1. Example search strategy from Scopus

| Major Search Term | Synonyms |
| --- | --- |
| Community | communit* OR “social network” OR “social support group” OR volunteer* OR neighbour OR neighbor OR “communit* group |
|  | AND |
| End of life | “end of life” OR death OR dying OR “palliative care” OR bereavement |
|  | AND |
| Work together | partnership* OR collaborat* OR ”teamwork” OR team* OR cooperat* OR “work together” |
|  | AND |
| Care | care OR caring OR caregiver |
